# Social Interaction with an “Unidentified Moving Object” Elicits A-Not-B Error in Domestic Dogs

**DOI:** 10.1371/journal.pone.0151600

**Published:** 2016-04-13

**Authors:** Anna Gergely, Anna B. Compton, Ruth C. Newberry, Ádám Miklósi

**Affiliations:** 1 Institute of Cognitive Neuroscience and Psychology, Hungarian Academy of Sciences, Budapest, Hungary; 2 Department of Animal Husbandry and Aquaculture, Norwegian University of Life Sciences, Ås, Norway; 3 Department of Ethology, Eötvös Loránd University, Budapest, Hungary; 4 MTA-ELTE Comparative Ethology Research Group, Budapest, Hungary; University of Lethbridge, CANADA

## Abstract

Mechanical “unidentified moving objects” (UMO’s) are useful for controlled investigations into features of social interaction that generate cooperativeness and positive social affiliation in domestic dogs (*Canis familiaris*). We hypothesized that, if a UMO interacted socially with a dog, the UMO would become associated with certain social cues and would subsequently affect dog behaviour. We assigned dogs to a Human, Social UMO or Non-Social UMO partner. In the Human and Social UMO conditions, the partner interacted with the dog cooperatively whereas the Non-Social UMO partner was unresponsive to the dog’s actions. We then tested dogs with their partner in a Piagetian A-not-B error paradigm, predicting that the Human and Social UMO partners would be more likely to elicit A-not-B errors in dogs than the Non-Social UMO partner. Five trials were conducted in which the dog watched its partner hide a ball behind one of two screens (A or B). As predicted, dogs in the Human and Social UMO conditions were more likely to search for the ball behind the A screen during B trials than dogs in the Non-Social UMO condition. These results reveal that the unfamiliar partner’s social responsiveness leads rapidly to accepting information communicated by the partner. This study has generated a better understanding of crucial features of agents that promote dog social behaviour, which will facilitate the programming of robots for various cooperative tasks.

## Introduction

Humans are adapted to efficiently acquire generalizable information through social learning. During development, cues signalling generalizable information acquire meaning. Csibra and Gergely [1] propose that human infants are especially receptive to this natural pedagogy, which may contribute to errors in the classic Piagetian A-not-B error paradigm. In this paradigm, originally developed to assess infant capacity to understand object permanence [2], an experimenter sitting opposite to an infant has two identical hiding spots (A and B) that the infant can reach. Over a series of trials, the experimenter visibly hides a desirable object under spot A or B, and the infant is then given the opportunity to search for and retrieve the object. The A-not-B error, which is common at around 8 to 10 months of age, refers to incorrect attribution of the hiding spot. Specifically, the infant persistently searches in spot A after observing the object disappearing under spot A but then re-emerging and being hidden under spot B [3]. Topál et al. [3] found that the likelihood of this perseverative A-not-B error increased if the experimenter used ostensive cues when showing the object being placed in each hiding spot. These cues included eye contact, mothereese, addressing the infant by name, and gaze alterations between the infant and the hiding spot. Instead of the cues providing episodic information that “this object is hidden under A or under B”, the infants appeared to interpret the cues as signalling generalizable information in the form of “objects like this can be found under A” [1, 3].

Extending their previous research, Topál et al. [4] reported similar use of cues by domestic dogs (*Canis familiaris*) in an A-not-B error paradigm. They compared three treatment groups. The Social Communicative (SocCom) group received ostensive cues from the experimenter, including vocal communication, referring to the dog by name, and gaze alterations between the dog and the hiding places. The Non-Communicative (NonCom) group received no ostensive cues; the experimenter conducted trials without eye contact or vocally addressing the dog. The Non-Social (NonSoc) group had no experimenter present. An “invisible” (transparent) string was attached to the desirable object (a ball) so the experimenter could move it to the two hiding spots from outside the room. Dogs in the SocCom group made more A-not-B errors in B trials than dogs in the NonCom group while, in the NonSoc group, errors ceased to occur. These findings strengthen support for the importance of ostensive cues in eliciting the A-not-B error.

Sümegi et al. [5] reported three main findings in a systematic examination of potential factors explaining dog responses in the A-not-B error task. Firstly, dog motivation to fetch the hidden object affected results; under-motivated dogs did not perform the task while over-motivated dogs seemed to ignore ostensive cueing and mostly chose the right spot. Secondly, human cueing performed in a communicative manner induced more errors than the same actions performed in a non-communicative manner. Thirdly, sham-baiting during the B-trials, whereby the experimenter gave ostensive cues at hiding spot A, and then at B, was important for eliciting the error. Sümegi et al. [5] suggested that the sham-baiting evoked a form of obedience, with the dog searching at the first place cued. This sham-baiting effect is consistent with findings in a two-choice food test (one bowl baited with food, one empty) in which dogs follow a human pointing gesture to the baited bowl [6, 7]. Szetei et al. [8] found that, in the absence of pointing, dogs chose the visibly baited bowl. However, dogs lost their preference when the experimenter pointed at the empty bowl even if they saw food being hidden in the other bowl. Being attentive to human communication and willing to cooperate with us, it seems that dogs prefer to rely on information cued by the experimenter and base their choices on it.

Social behaviour is difficult to study due to lack of full control over the interaction. This is true even when studying human-animal interactions. As humans, we assume that we can control our behaviour but components of human behaviour below conscious awareness may serve as sources of bias in behavioural studies [9]. Therefore, the use of robotic agents has become popular as a tool for teasing out the effects of different aspects of social interaction [10], and successfully implemented in studies focused on dogs’ social cognition [11, 12, 13, 14, 15].

To examine the role of agent responsiveness, Gergely et al. [14] assigned dogs to one of three partner types, a Social “unidentified moving object” (UMO), a Mechanical UMO, or a Mechanical Human, for a problem-solving task involving a food reward that was unattainable by the dog without assistance from the partner. The two mechanical partners differed in appearance but acted in a similarly unresponsive manner to the dog. The Social UMO, on the other hand, was responsive to the dog’s behaviour. Dogs spent the most time looking at their partner, and alternated their gaze between the unobtainable food and the partner most frequently, when partnered with the Social UMO. In a subsequent study, it was found that dogs were able to find hidden food based on a UMO’s directional signal immediately after a brief social interaction with the agent [15]. These results suggest that the interactive behaviour of an agent is important in eliciting dogs’ social behaviour and also facilitates the recognition of potentially communicative actions [14, 15].

We further explored the role of partner responsiveness in the current study, assessing whether experience with an interactive social partner would affect the salience of ostensive cues given by the same partner in the A-not-B error paradigm. We hypothesized that an agent’s sociality would play a role in producing errors by dogs in the A-not-B task. We assigned dogs to three partner types varying in level of interactivity with the dog, a Non-Social UMO (negative control), Social UMO (experimental), or Human (positive control). The Social UMO and Human partners interacted socially with the dog using ostensive cues in familiarisation sessions, whereas the Non-Social UMO was non-interactive. We predicted that dogs would perform more A-not-B errors if they had been paired with the Social UMO or Human partner than with the Non-Social UMO partner. We expected that this study would improve basic understanding of social behaviour, while also having practical relevance to the programming of robots for various cooperative tasks with dogs.

## Materials and Methods

### Ethics statement

Our study was conducted at Eötvös Loránd University in Budapest, Hungary, where animal experiments are overseen by the University Institutional Animal Care and Use Committee (UIACUC). The currently operating Hungarian law ‘‘1998. évi XXVIII. Törvény”—the Animal Protection Act—defines experiments on animals in the 9^th^ point of its 3^rd^paragraph (3. 1/9.). According to this definition, our non-invasive observational experiment on dog behaviour was not considered as an animal experiment under the law and was therefore allowed to be conducted without any special permission from the UIACUC.

All participation by owners with their dogs was voluntary, and owners stayed with their dog during the study. The owners filled out a consent form to permit their dogs to participate in the study, and to allow use of the resulting media in publications. They were informed that the study was about canine learning, and told about the procedure, but were blind to the hypothesis and predictions.

### Subjects

Sixty-two adult family dogs were recruited through the database for volunteers to the Family Dog Project at Eötvös Loránd University. Any dogs with previous experience of A-not-B error tasks were excluded. Because the study required motivation to gain access to a hidden tennis ball, we pre-tested dogs for interest in tennis balls (see Phase 1 below). Using the scoring system of Sümegi et al. [5], we excluded 7 under-motivated dogs (showing no interest in the ball or in searching for it) and 7 over-motivated dogs (repeatedly lunging for the ball when the experimenter or owner was holding it, trying to break loose to get the ball, refusing to release the ball). The remaining 48 dogs (32 purebred dogs from 20 different breeds and 16 mixed-breed dogs) were divided among three experimental conditions: Human N = 16, Social UMO N = 16 and Non-social UMO N = 16 (see S1 Table). They ranged from 1 to 11 years of age, with a mean age of 5.1 years (S.E. ± 3 years). Half of the subjects had already participated in previous studies in which a social UMO helped the dog to obtain an unreachable food reward (see [14, 15]). Therefore, randomisation across the experimental conditions was performed separately for dogs with and without this prior social experience (see S1 Table).

### Apparatus

**Fig 1:**
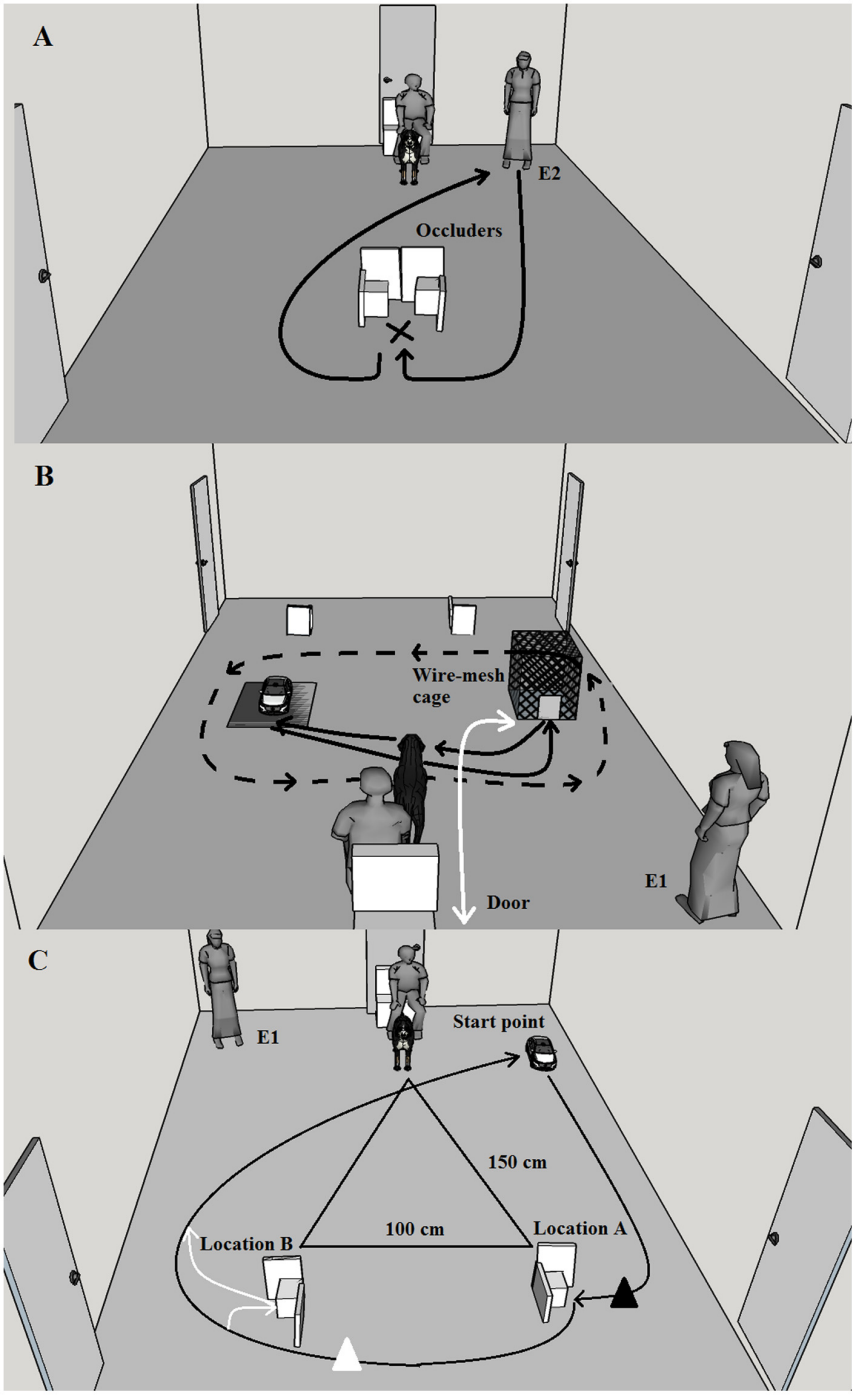
Experimental layout. (A) Phase 1: The x indicates the location of the hidden ball behind the white occluders. Black lines show the path of Experimenter 2 (E2) during the hiding procedure while the owner and dog sat watching. (B) Phase 2: In the Non-social condition, the dashed black line indicates the path of the remote-controlled car (UMO) operated by E1. In the Social UMO condition, the white line indicates the route used by E2 while entering the room, putting down the wire-mesh cage, attaching the tray with the ball inside the cage, then leaving the room. The black lines show the route of the Social UMO. The locations of the wire-mesh cage and Social UMO were alternated in successive trials. (C) Phase 3: The UMO location represents the UMO (or Human) starting point. Black lines show the path of the UMO (or Human) partner to location A, and around to the start point, during A trials. The black triangle indicates the signal spot (beeping/“Look” signal given by the partner) for location A. White lines show the path of the partner (Human or UMO) in B trials when making an additional visit to the B occluder. The white triangle indicates the signal spot (beeping/“Look” signal given by the partner) for location B.

The experiment was conducted in a laboratory room (5 m x 2.5 m) with tape on the floor marking standardized locations and pathways to be used in each phase of the experiment (Fig 1). A video camera was mounted on each wall, with output recorded on computer. Two occluders, used for hiding a yellow tennis ball from the dogs, were placed 50 cm from the front wall of the room. Each occluder was constructed from two white plastic boards (40 x 25 x 0.2 cm) glued to a brick (13 x 17 x 18 cm). One board was set upright (40 cm high) facing towards the back of the room, and the second board was placed on its side (25 cm high) at right angles to the first. The two occluders were placed together during Phase 1 of the experiment, and 100 cm apart from each other (50 cm out from each side wall) during Phases 2 and 3 (Fig 1).

The UMO (Traxxas Ford Mustang Boss #302 remote-controlled car, dimensions: 37 x 19 x 12 cm) was fitted with a plastic tray (6.5 x 6.5 x 2 cm) which was used to transport the ball for dogs in the UMO conditions during Phases 2 and 3. The tray had a metal sheet on each end, one for attaching it to a magnet on the UMO and the other for attaching it to strong magnets on the bricks behind the occluders in Phase 3 (see Procedure). In the Social UMO condition, a metal wire-mesh cage (61 cm x 46 cm x 54 cm) with a small door was used in Phase 2. It had a fixed magnet inside to which the tray with the ball could be weakly attached (see Fig 1B and Procedure).

### Procedure

The experiment was conducted in three phases, with dogs being exposed to each phase consecutively. Phase 1 (Pre-test) was identical for all dogs. Phase 2 (Familiarisation) differed between the three conditions. In Phase 3 (Test), five trials of the A-not-B error test were conducted. Dogs were given a brief, 10-s break between phases. A stopwatch was used to standardise the duration of Phases 1 and 2 to 2 min. In Phases 2 and 3, Experimenter 1 (E1) stood quietly in one corner to operate the UMO with a remote controller. Experimenter 2 (E2) served various functions (described below).

#### Phase 1 (pre-test)

The owner and dog entered the room and the dog could sniff and explore the room for 1 min. Then the owner sat down on a chair placed in a predetermined location and held the dog in front of him/herself. E2 took the ball, showed it to the dog, walked behind the occluders (which together formed one hiding spot, see Fig 1A), and attracted the dog's attention by calling the dog’s name and saying “Look here”. E2 then placed the ball behind the occluders, walked back to the dog showing her empty hands, and went back to the starting point. The dog was then released and given verbal encouragement by the owner to find and retrieve the ball (e.g. “Go!”, “Get the ball!”). This process was repeated twice during this 2-min phase.

#### Phase 2 (familiarisation)

The owner sat in the chair. Dogs in the Human condition received 2 min of ball play with E2, who moved around the room calling the dog’s attention and throwing the ball for the dog from different directions. For dogs in the Non-Social UMO treatment, the owner held the dog in front of him/herself while the UMO circled around the room for 2 min (see Fig 1B). On its first time past the dog, the UMO stopped to allow the dog to sniff it. If the dog showed signs of anxiety, the UMO kept further away from the dog.

For dogs in the Social UMO condition, the owner held the dog in front of him/herself. E2 entered the room with the wire-mesh cage and placed it in its predetermined location (Fig 1B). Then she called the dog’s attention by calling the dog’s name and saying “Look here”, then attached the tray with the ball to the magnet inside the cage and left the room through the door behind the owner’s chair (Fig 1B). The owner released the dog. As soon as the dog directed attention to the UMO (i.e. oriented toward the UMO or touching it with its muzzle), the UMO started to move towards the wire-mesh cage, entered the cage through a small door, attached to the tray with the magnet, beeped twice, and pulled the tray with the ball out of the cage making the ball accessible to the dog. If the dog did not look at the UMO within 15 s, the UMO beeped twice to attract the dog’s attention before driving to the cage. The owner praised the dog for retrieving the ball while the UMO returned to the starting point. E2 then entered the room, switched the location of the cage and the UMO (Fig 1B) and placed the ball and tray back into the cage. The sequence was repeated a total of six times which lasted a total of 2 min, with the cage location and the UMO’s starting point being alternated between left and right sides of the room. At the end of Phase 2, the owner left the room with the dog for 10 s in all conditions.

#### Phase 3 (test)

The owner and the dog entered the room, then the owner sat down at on the chair and held the dog in front of him/herself. Five trials were conducted in which the ball was hidden behind the left (A) or right (B) occluder in the sequence, A, A, B, B, A (Fig 1C). We included only two initial A trials and two B trials to minimise perseveration (i.e. a training effect [16]), and we included a final A trial to establish whether searching at location A would be maintained after the B trials [17]. Testing ended after five trials to avoid tiring and loss of attention by the dogs. In the Human condition, E2 played the role of the human partner whereas the UMO was the partner in the Social UMO and Non-social UMO conditions. The UMO behaved exactly the same way in both of the UMO conditions in this phase.

In A trials, the partner (Human, or UMO with tray holding ball attached) called the dog's attention at the starting point (Human: calling the dog’s name and saying “Look here”, UMO: beeped twice) and approached occluder A while carrying the ball visible to the dog. Then the partner (Human or UMO) stopped by A, holding the ball visible, and called the dog's attention (Human: calling the dog’s name and saying “Look here”, UMO: beeping sound). The partner (Human or UMO) then deposited the ball behind the occluder (with the UMO attaching the tray to a magnet on the brick and pulling loose), moved around B and returned to the start point (Fig 1C). After 3 s, the owner released the dog to search for the ball, with no signals (i.e. verbal or pointing) being given to the dog. After the dog found the ball, the owner called the dog back and gave praise while recovering the ball.

In B trials, the same steps were followed but, after stopping with the ball behind A, the partner (Human or UMO) moved to B still carrying the ball, stopped by B with the ball visible and called the dog's attention (Human: calling the dog’s name and saying “Look here”, UMO: beeped twice), and then deposited the ball behind B before returning to the start point (Fig 1C). In all trials, dogs were allowed to search until they found the ball even if they failed to find it behind the occluder in their first choice.

### Data collection

All trials were videotaped, and dog behaviour during Phase 3 (test phase) was analysed with Solomon Coder 090913 (András Péter, http://solomoncoder.com) (Table 1). The dog’s *choice* during the A-not-B trials was noted by E1 (i.e. live coding) after each trial and later confirmed from video recordings. The *Latency* to make a choice was included to gain insights into how the dogs were making their choices. *Looking at the partner* and *Touching the partner* were evaluated to assess whether these behaviours would be directed to the Human and Social UMO more often than the Non-social UMO.

**Table 1:** Ethogram listing definitions of the coded behavioural variables in Phase 3.

Variable name	Definition
Choice (binary)	For each test trial, if the first occluder behind which the dog put its head was the location of the hidden ball, the trial was scored as correct (score 1); if the other location was the first to be investigated, the trial was scored as incorrect (score 0)
Latency (duration)	Time span (s) from release by owner until placing head behind an occlude
Looking at the partner (duration)	Duration (s) spent with head oriented towards partner, from release by owner until placing head behind an occluder
Touching the partner (duration)	Duration (s) spent touching partner with muzzle, from release by owner until placing head behind an occlude

One dog was excluded from the study due to a car driving failure during testing, and video data for two dogs were missing due to video recording failure (see S1 Table). Thus, only data from the live coding were available for these latter two dogs (i.e. *Choice* variable). Inter-observer agreement (between two coders) was assessed by recoding the behaviour of a randomly-selected 10% of the subjects. The index of concordance was 100% for binomial variables and >90% for durations. In case of unresolved disagreement, coding by E1 was used.

### Statistical analysis

To control for repeated measures, we applied random intercept generalized linear mixed-effect models (GLMMs) to the data (see S2 Table) using IBM SPSS 21, with dog ID (dog’s name) included as a random grouping factor. For all variables, the fixed explanatory variables included condition, test trial and condition x test trial interaction. The *Choice* (binary) variable was analysed by GLMM with binomial distribution, *Latency* (duration) with Poisson distribution, and *Looking at the partner* (duration) and *Touching the partner* (duration) with Gaussian error distribution after logarithmic transformation of the response. The binary model was not over-dispersed. All tests were two-tailed and the α value was set at 0.05. Sequential Sidak correction was applied in all post-hoc comparisons.

First, we examined whether condition influenced *Choice* and *Latency* during the test trials in a model including condition (factor with 3 levels) and test trial (factor with 5 levels). In the case of the *Choice* variable, dog performance in each trial was also compared to chance level (0.5) with the one-sample binomial test with false discovery rate (FDR) correction. Third, we examined whether condition influenced dog behaviour towards the partner during test trials. Since none of the dogs looked at or touched the partner in the Human condition after release to search for the ball, we included only the Social and Non-social UMO conditions in a model examining effects of condition (factor with 2 levels) and test trial (factor with 5 levels). Since the condition by trial interactions were not significant (*Looking at the partner*: F_4,72_ = 0.74, p = 0.57; *Touching the partner*: F_4,52_ = 0.8, p = 0.53), they were removed from the models.

## Results

In general, dogs made clear choices. When released to find the ball, they headed directly towards one occluder and put their head behind it. In three trials, the dog either started to move towards one occluder and then changed direction, or moved towards one occluder but then, without looking behind it, kept going to the other occluder. Their choices were registered based on the occluder that they first put their head behind, according to the established criterion.

**Fig 2:**
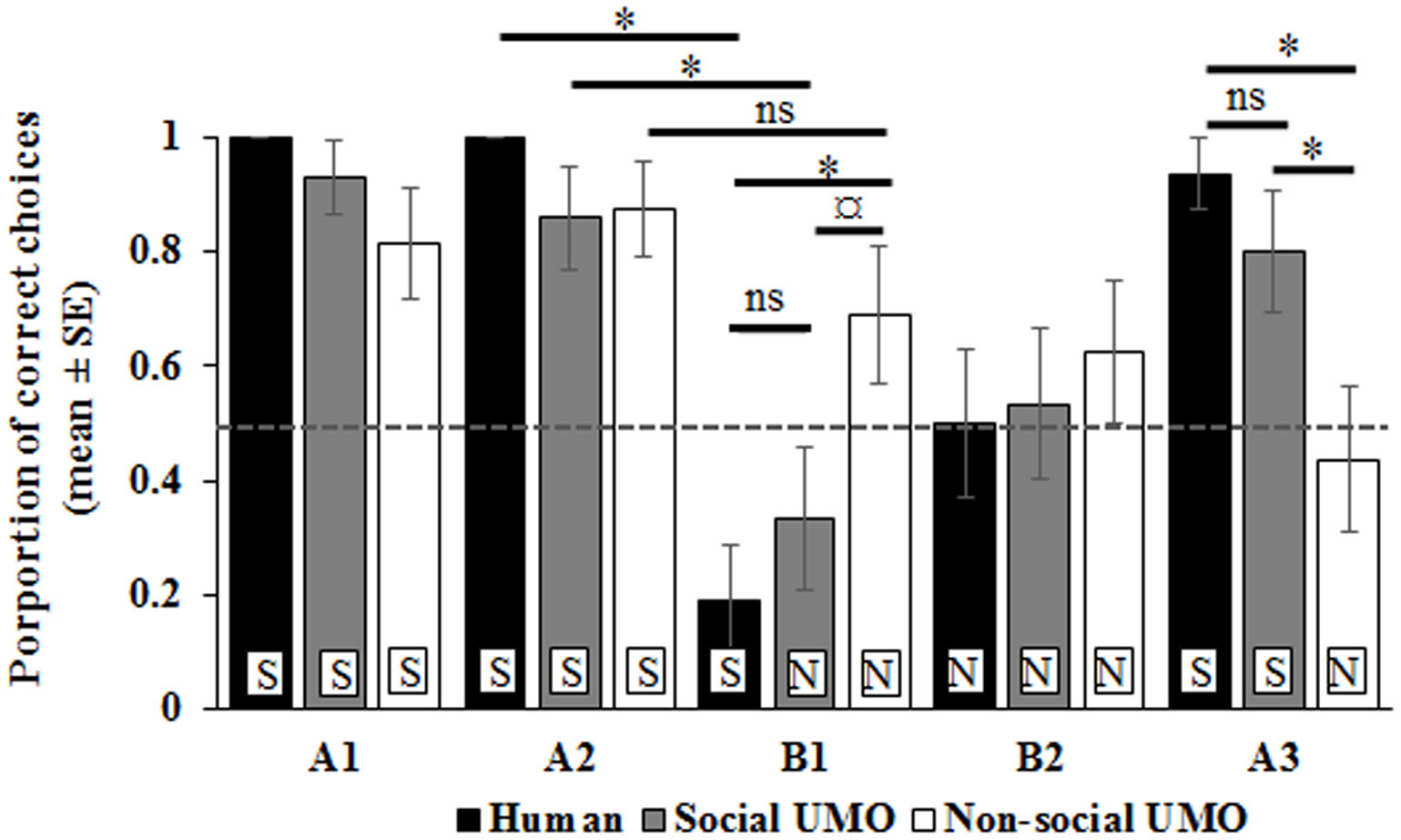
Dog accuracy in finding the hidden ball at location A or B during test trials. Dogs in three conditions were tested in five successive trials (A1, A2, B1, B2, A3; letter denotes hiding spot). Dotted line shows chance level (0.5), while capital letters mark comparisons to chance level (S: p<0.05; N: p>0.05). * p≤0.05 ¤ p = 0.07 ns p>0.08 for condition within trial.

In the binomial tests, our results showed that dogs chose the correct hiding place above chance level in A1 and A2 trials in all conditions (A1: all conditions p≤0.02; A2: all conditions p≤0.004). In sharp contrast, during the B1 trial their performance decreased below chance level in the Human condition (p = 0.021) or occurred at chance level in the Social (p = 0.45) and Non-social UMO (p = 0.21) conditions (Fig 2). In the second B trial, dogs chose randomly between hiding places in all conditions (B2: all conditions p>0.05). However, during the final, A3 trial, dogs in the Human (p = 0.001) and Social UMO (p = 0.021) conditions performed above chance, while dogs in the Non-social UMO condition stayed at chance level (p = 0.8).

Based on the results of the GLMM, we found a significant condition * test trial interaction (F_8,220_ = 2.17, p = 0.031). Dog performance did not differ between conditions during the A1, A2 and B2 trials (A1: F_2,220_ = 2.28, p = 0.1; A2: F_2,220_ = 2.21, p = 0.11; B2: F_2,220_ = 0.27, p = 0.77), whereas significant differences were found in the B1 and A3 trials (B1: F_2,220_ = 5.35, p = 0.005; A3: F_2,220_ = 6.3, p = 0.002). Pairwise comparisons showed that dogs in the Human condition made more errors during the B1 trial compared to dogs in the Non-social UMO condition (p = 0.004) while only a marginal difference was found between the Social and Non-Social UMO condition (p = 0.07) and no difference was found between the Human and Social UMO condition (p = 0.36). Dogs’ accuracy in the A3 trial was lower in the Non-social UMO condition compared to the other conditions (Non-Social UMO vs. Human: p = 0.002; Non-Social UMO vs. Social UMO: p = 0.05) while no difference was found between the Human and Social UMO condition (p = 0.26) (Fig 2).

Pairwise comparisons of trials within conditions revealed that dog performance in A trials differed from that in the B1 trial in the Human and Social UMO conditions but not in the Non-social UMO condition where only the last A trial differed from the second A trial (see Fig 2 and Table 2).

**Table 2:** Results of pairwise comparisons of test trials (adjusted p values from the Binomial GLMM with Sequential Sidak correction).

Test trials	Human	Social UMO	Non-social UMO
A1–A2	1.00	0.54	0.86
A1–B1	**<0.01**	**<0.01**	0.79
A1–B2	**<0.01**	**0.04**	0.73
A1–A3	0.67	0.69	0.15
A2–B1	**<0.01**	**<0.01**	0.72
A2–B2	**<0.01**	0.18	0.53
A2–A3	0.67	0.79	**0.03**
B1–B2	0.19	0.69	0.86
B1–A3	**<0.01**	**0.03**	0.65
B2–A3	**= 0.01**	0.43	0.73

Low performance during the A3 trial in the Non-Social UMO condition could have been due to reward history (i.e. history of reinforced responses). It has been shown that infants perseverate more following a correct response than following an incorrect response (e.g. [18]) and, thus, successful search at the B location could explain the higher error rate in the last A trial in the present study. To assess the weight of evidence for this hypothesis, we categorised dogs based on the number of their successful/unsuccessful searches during the first four trials (AABB). We found that 50% of the Non-social UMO subjects successfully switched from A to B for both B trials after 100% success in the first two A trials whereas only 12.5% of the subjects in the Human and Social UMO conditions were equally successful (Table 3).

**Table 3:** Percentage of subjects in each reward history category by condition.

	Reward History Categories	Human	Social UMO	Non-social UMO
	(1) 0 error at A 2 errors at B	43.75%	31.25%	18.75%
Perseverated	(2) 0 error at A 1 error at B	43.75%	37.5%	12.5%
	**Total**	**87.5%**	**68.75%**	**31.25%**
	(3) 0 error at A 0 error at B	12.5%	12.5%	50%
Did not perseverate	(4) alternating strategy (1 or 2 errors at A with 1 or 2 errors at B)	0%	18.75%	18.75%
	**Total**	**12.5%**	**31.25%**	**68.75%**

**Fig 3:**
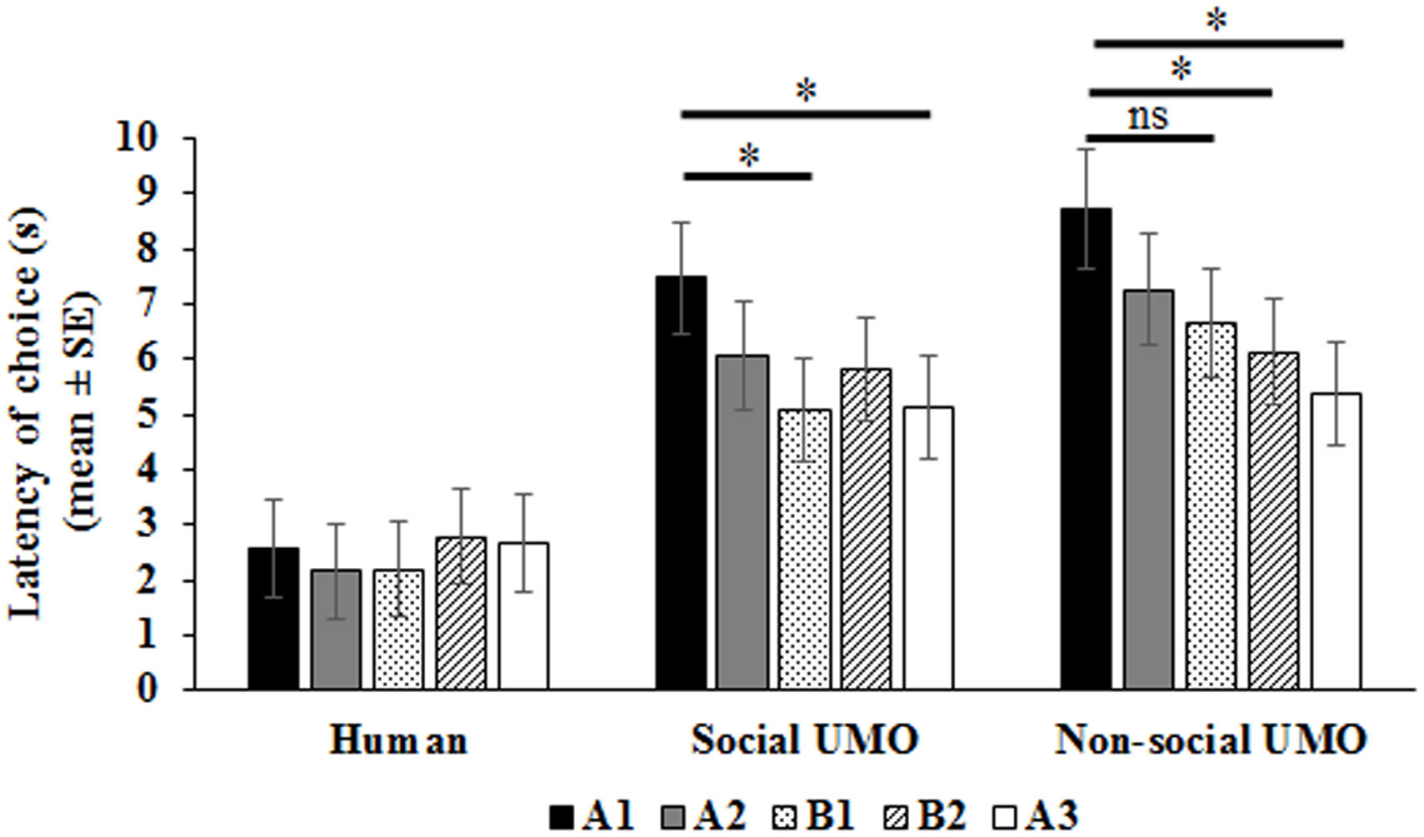
Latency of choice during test trials. * p<0.05; ns p>0.05 for trials within condition.

Analysis of *Latency* revealed a significant condition * trial interaction (F_8,210_ = 2.33, p = 0.02). In the Human condition, latency to choose did not differ between trials (F_4,210_ = 0.61, p = 0.65) whereas differences were found for the two UMO conditions (Social UMO: F_4,210_ = 2.77, p = 0.03; Non-social UMO: F_4,210_ = 4.25, p = 0.002). Pairwise comparisons showed that dogs in the Social UMO condition made their choice faster in the A1 trial compared to B1 (p = 0.03), and A3 trials (p = 0.04). In the Non-social UMO condition, the latency of choice did not differ between the A1 and B1 trials (p = 0.16) but did differ between A1 and B2 (p = 0.04), and A1 and A3 (p = 0.001) (Fig 3). Latencies did not differ between the two UMO conditions across trials (p>0.1), but did differ from those in the Human condition across all trials (p≤0.03). We can assume that the increased latencies in the two UMO conditions were due to performance of other behaviours (i.e. looking and touching the partner) that preceded choice and that were missing in the Human condition.

Finally, we investigated dogs’ looking (i.e. look at the UMO) and touching behaviour (i.e. touching the UMO) in the two UMO conditions following release in the test trials (when none of the dogs looked at or touched the human partner). Our results showed that none of the factors (i.e. condition and trial) affected dogs' behaviour in the Social and Non-Social UMO condition (*Looking at the partner*: condition F_1,76_ = 0.33, p = 0.57, trial: F_4,76_ = 1.67, p = 0.17; *Touching the partner*: condition F_1,56_ = 0.98, p = 0.32, trial: F_4,56_ = 0.4, p = 0.8).

## Discussion

The goal of this research was to investigate the experience needed for a dog to recognise aspects of an artificial agent’s behaviour as being of relevance. The A-not-B error paradigm was used to assess whether dogs would be more responsive to the ostensive cues of their partner if the partner had interacted with them in a cooperative manner. The partners varied in sociality during the familiarisation phase, with the Human and Social UMO partners being cooperative in providing access to the ball whereas the Non-Social UMO’s behaviour was unresponsive. Consequently, we predicted that dogs would rely more on the cues given by the Human and Social UMO agents than the Non-Social UMO agent in the A-not-B error trials, resulting in more errors in the B trials compared to A trials. This prediction was based on two crucial findings in previous studies. Firstly, human agents using ostensive cues elicited more errors by dogs in B trials than human agents that did not give such cues [4]. Therefore, all agents in the current study gave ostensive cues during hiding of the ball in the A-not-B error trials. Secondly, dogs interacted more with a UMO by looking at it more, and showing more gaze alternations between it and inaccessible food, if it was cooperative in helping the dog gain access to food than if it moved in a mechanical, unresponsive manner [14, 15]. Therefore, in the current study, we investigated whether such social experience, this time to gain access to a ball rather than food as the reward, would have a carry-over effect on responses in the A-not-B error test.

The results support our prediction. Dogs in the Non-Social UMO treatment condition did not commit the A-not-B error, since their performance did not decrease significantly from A to B trials [3], as predicted if they did not consider the ‘beep’ cues conveyed by the UMO as having relevance. They responded similarly to dogs with a non-communicative human, or no partner (ball moved by ‘invisible’ string) in previous work [4]. In contrast, dogs responded to the beeps of the Social UMO, and even more to the “Look” cues of the Human partner, in a manner suggesting that they relied on information from their partner and utilised it in making their choice in the A-not-B error trials. From these findings, taken together with the observations of Kubinyi et al. [11] and Gergely et al. [14, 15], it appears that social behaviour plays an important role in determining a dog’s willingness to engage in affiliative interactions with agents, despite differences in embodiment to their typical canine and human social companions.

The high error rate of dogs in the Human condition in B trials is consistent with the results of Topál et al. [4] for dogs and infants responding to human ostensive communication in B trials, attesting to the soundness of our Human condition as a positive control group against which to compare the two UMO conditions. Dogs’ performance in the Human and Social UMO conditions followed the same pattern as found in a recent study using the same sequence of trials (i.e. AABBA) with a human partner [17]. However, decreased search accuracy observed in the Non-social UMO condition at location A after B trials was not found previously. We can assume that the dogs’ decreased performance in A3 may have been influenced by their relatively high success in B trials leading to maintained search activity at location B (see reward history in Table 3). This win-stay, lose-shift strategy has been shown in several studies focused on spatial memory and searching behaviour in human and non-human animals [18, 19, 20, 21] including dogs (e.g. [22]). As an alternative explanation, other factors such as attention and fatigue can also cause decreased performance across multiple hiding events in dogs, similarly to other species (e.g. common marmosets [23]).

In summary, dogs in the present study rapidly established expectations about the behaviour of the UMO and based subsequent behaviour upon these expectations. Consistent with the concept of convergent social evolution between humans and dogs [4], the dogs’ sensitivity to novel social partners provides evidence of their developmental behavioural plasticity, facilitating their adaptation to the human environment.

A potential application of understanding how dogs interact with artificial agents involves humans, dogs and robots cooperating to solve tasks. For example, in Search and Rescue (SAR) operations, drones could be used to search areas and interact with SAR dogs [24]. To follow a drone (i.e., respond to its “commands”), the dog would have to consider the information conveyed by the drone as having importance. The current work has generated a better understanding of crucial features of agents that promote dog social behaviour, which will facilitate the programming of robots for various cooperative tasks.

## Conclusions

Gergely et al. [14] were the first to present evidence of the importance of interactive behaviour of an artificial agent in evoking canine social responsiveness. The current study builds upon that work, showing that dogs can rapidly form expectations about an agent’s behaviour and utilize this understanding in subsequent interactions. This study shows that the quality of interaction with an agent immediately before testing is an important factor in determining whether or not the agent’s behaviour will evoke A-not-B error in dogs.

## Supporting Information

S1 Table Detailed information about the subjects. (XLSX)

S2 Table Data file. (XLSX)
